# Polyclonal Spread of Fosfomycin Resistance among Carbapenemase-Producing Members of the *Enterobacterales* in the Czech Republic

**DOI:** 10.1128/spectrum.00095-23

**Published:** 2023-04-26

**Authors:** V. Mattioni Marchetti, L. Kraftova, M. Finianos, T. Sourenian, J. Hrabak, I. Bitar

**Affiliations:** a Department of Microbiology, Faculty of Medicine, University Hospital in Pilsen, Charles University, Pilsen, Czech Republic; b Biomedical Center, Faculty of Medicine, Charles University, Pilsen, Czech Republic; Instituto de Higiene

**Keywords:** *Enterobacterales*, carbapenemase producers, fosfomycin, drug-resistance bacteria, WGS

## Abstract

Fosfomycin (FOS) has been recently reintroduced into clinical practice, but its effectiveness against multidrug-resistant (MDR) *Enterobacterales* is reduced due to the emergence of FOS resistance. The copresence of carbapenemases and FOS resistance could drastically limit antibiotic treatment. The aims of this study were (i) to investigate fosfomycin susceptibility profiles among carbapenem-resistant *Enterobacterales* (CRE) in the Czech Republic, (ii) to characterize the genetic environment of *fosA* genes among the collection, and (iii) to evaluate the presence of amino acid mutations in proteins involved in FOS resistance mechanisms. During the period from December 2018 to February 2022, 293 CRE isolates were collected from different hospitals in the Czech Republic. FOS MICs were assessed by the agar dilution method (ADM), FosA and FosC2 production was detected by the sodium phosphonoformate (PPF) test, and the presence of *fosA-*like genes was confirmed by PCR. Whole-genome sequencing was conducted with an Illumina NovaSeq 6000 system on selected strains, and the effect of point mutations in the FOS pathway was predicted using PROVEAN. Of these strains, 29% showed low susceptibility to fosfomycin (MIC, ≥16 μg/mL) by ADM. An NDM-producing Escherichia coli sequence type 648 (ST648) strain harbored a *fosA10* gene on an IncK plasmid, while a VIM-producing Citrobacter freundii ST673 strain harbored a new *fosA7* variant, designated *fosA7.9*. Analysis of mutations in the FOS pathway revealed several deleterious mutations occurring in GlpT, UhpT, UhpC, CyaA, and GlpR. Results regarding single substitutions in amino acid sequences highlighted a relationship between ST and specific mutations and an enhanced predisposition for certain STs to develop resistance. This study highlights the occurrence of several FOS resistance mechanisms in different clones spreading in the Czech Republic.

**IMPORTANCE** Antimicrobial resistance (AMR) currently represents a concern for human health, and the reintroduction of antibiotics such as fosfomycin into clinical practice can provide further option in treatment of multidrug-resistant (MDR) bacterial infections. However, there is a global increase of fosfomycin-resistant bacteria, reducing its effectiveness. Considering this increase, it is crucial to monitor the spread of fosfomycin resistance in MDR bacteria in clinical settings and to investigate the resistance mechanism at the molecular level. Our study reports a large variety of fosfomycin resistance mechanisms among carbapenemase-producing *Enterobacterales* (CRE) in the Czech Republic. Our study summarizes the main achievements of our research on the use of molecular technologies, such as next-generation sequencing (NGS), to describe the heterogeneous mechanisms that reduce fosfomycin effectiveness in CRE. The results suggest that a program for widespread monitoring of fosfomycin resistance and epidemiology fosfomycin-resistant organisms can aide timely implementation of countermeasures to maintain the effectiveness of fosfomycin.

## INTRODUCTION

Fosfomycin (FOS) is a phosphoric acid derivate, active against both Gram-negative and Gram-positive bacteria. It was discovered in 1969, and it gained renewed clinical interest in the last 20 years as a good candidate in the treatment of multidrug-resistant (MDR) bacterial infections (1). According to the Food and Drug Administration (FDA) and the European Medicine Agency (EMA), FOS is approved for oral use in uncomplicated lower urinary tract infections (UTI) and for systemic use in complicated UTI and bacterial meningitis (https://www.accessdata.fda.gov/drugsatfda_docs/label/2008/050717s005lbl.pdf). FOS binds to UDP-*N*-acetylglucosamine enolpyruvyl transferase (MurA), which interferes with the early stages of peptidoglycan production (2). FOS uptake inside bacterial cell depends on two transport systems: the glycerol-3-phosphate transporter (GlpT) and the glucose-6-phosphate transporter (UhpT). The expression of GlpT and UhpT is glycerol-3 (G3P) and glucose-6-phosphate (G6P) dependent and requires the presence of cyclic AMP (cAMP). Moreover, UhpT expression is further controlled by the UhpABC system (2). Inactivation of UhpABC impairs expression levels of UhpT and affects FOS uptake into the cytosol (3–8).

**FIG 1:**
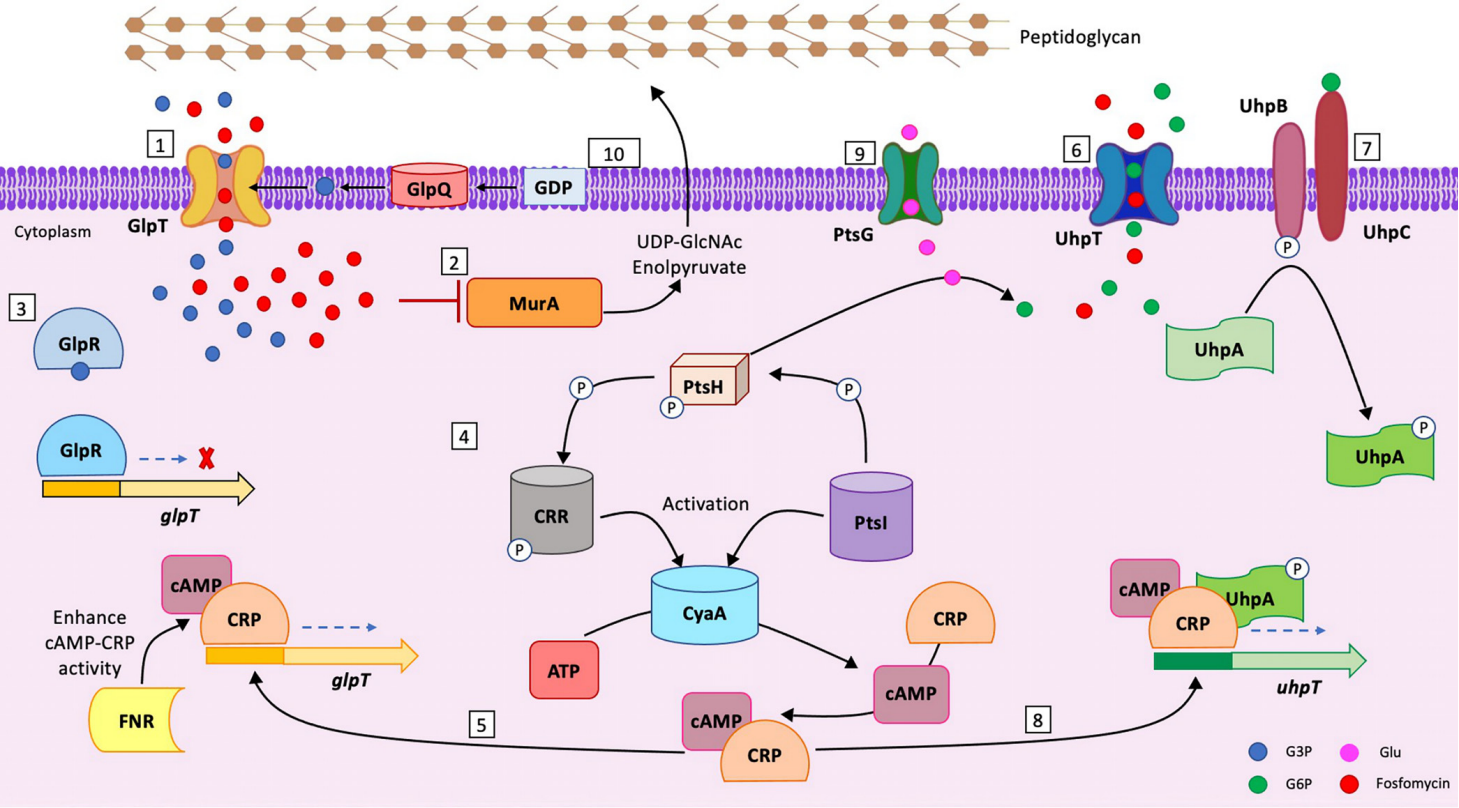
FOS uptake and pathway inside the bacterial cell. (Step 1) GlpT transports G3P and fosfomycin inside the bacterial cell. GlpT expression is mediated by G3P. (Step 2) Inside the cytoplasm, FOS binds to MurA and interferes with the formation of UDP-GlcNAc enolpyruvate, the first stage of peptidoglycan biosynthesis. (Step 3) GlpR acts as a repressor for GlpT expression. In the cytoplasm, G3P attaches to GlpR, blocking its binding to *glpT* promoter. (Step 4) PtsI transfers a P group from PEP (2-phosphoenolpyruvate) to PtsH. The P group is then transferred to CRR by PtsH. CRR-P and PtsI activate CyaA (4). CyaA is an adenylate cyclase that converts the ATP to cAMP. cAMP binds to the CRP, and the cAMP-CRP complex promotes expression of both GlpT and UhpT (5). (Step 5) cAMP-CRP complex promotes GlpT expression, binding to *glpT* promoter. The activity of the cAMP-cAMP receptor protein (CRP) complex is enhanced by FNR (6). (Step 6) UhpT promotes the entry of G6P and FOS into the cell. The presence of G6P enhances the expression levels of UhpT. (Step 7) The UhpABC system promotes the expression of UhpT. UhpC binds extracellular G6P, and through UhpB, a phosphate group is transferred to UhpA (UhpA-P). (Step 8) UhpA-P is the activate form of UhpA and, together with cAMP-CRP complex, starts UhpT transcription, binding to the *uhpT* promoter. (Step 9) PtsG promotes the entry of glucose into the cell (7). (Step 10) GlpQ is a periplasmic glycerophosphodiester phosphodiesterase that converts periplasmic glycerophosphodiesters to G3P (8).

The levels of cAMP are directly related to the adenylate cyclase CyaA and to the phosphotransferase PtsI. The occurrence of mutations in both CyaA and PtsI alters cAMP intracellular levels and, consequently, FOS uptake (9) (Fig. 1).

Several mutations can take place at different steps of the above-mentioned pathway, affecting FOS uptake and leading to different extents of FOS resistance (2, 10, 11). Alteration of GlpT/UhpT activity and mutations in MurA are considered the primary FOS resistance mechanism, followed by impairment in UhpA, CyaA, and PtsI activity (9) (Fig. 1).

In recent years, a great deal of attention has been given to FOS-modifying enzymes, such as FosA. FosA is a metalloenzyme that catalyzes the opening of the FOS epoxide ring, leading to its inactivation (12). As of December 2022, 10 *fosA* variants have been reported on both chromosomes and plasmids in members of the *Enterobacterales*. *fosA*-like genes are chromosomally distributed in *Providencia* spp. and Serratia marcescens (*fosA*), in Enterobacter spp. (*fosA2*), in Salmonella species (*fosA7*), and in Klebsiella pneumoniae (*fosA5* and *fosA6*) (12). In contrast, plasmid-mediated dissemination is recognized for *fosA3*, *fosA4*, *fosA8*, *fosA9*, and *fosA10* among Escherichia coli and K. pneumoniae strains (13–16). Epidemiologically, FosA3 is the most widespread variant, with endemic cases reported from both veterinary and clinical settings in China (13, 17–19); FosA7 is predominantly found in isolates from veterinary settings (20, 21). FosA5 has been reported from clinical settings in China (22, 23) and from food in India (24), while FosA10 has been detected only in chicken meat from China (16). Few cases from food and clinical samples reported the occurrence of FosA8 (25, 26), while only one clinical case involving FosA9 has been described from a clinical case in the Netherlands (27). The co-occurrence of FosA enzymes and extended-spectrum β-lactamases (ESBLs), including carbapenemases, in *Enterobacterales* has been already reported in the literature, limiting the use of FOS in clinical practice (28, 29). Co-occurrence of FosA enzymes and carbapenemases has been mainly described for K. pneumoniae (17, 28, 30), with sporadic occurrences in E. coli (31–33) and two reports of occurrences in Citrobacter freundii (34).

Escherichia coli is an intestinal commensal of warmed-blood animals that has the ability to cause diseases such as extraintestinal infections (13). *Citrobacter* spp. are low-risk pathogens but, as reported in the literature, can act as a silent reservoir for relevant resistance genes, especially C. freundii (35). The occurrence of FosA enzymes has been documented worldwide, but large epidemiological investigations of their occurrence in E. coli and in *Citrobacter* spp. are lacking, especially among European countries. The Czech Republic has an active and broad surveillance program for carbapenem-resistant *Enterobacterales* (CRE) among clinical settings (36, 37), but no epidemiological investigation of FOS resistance among these strains has been conducted recently. Moreover, as reported in previous studies, the Czech Republic has an increasing number of cases of disease caused by carbapenemase-producing E. coli and C. freundii, and the occurrence of fosfomycin resistance mechanisms in such isolates represents a concerning public health issue (35–37).

The aim of our study was to characterize the epidemiology of FOS resistance in the Czech Republic clinical setting in E. coli and *Citrobacter* carbapenemase producers. Moreover, an additional aim was to characterize the resistance mechanisms of FOS resistance through the detection of *fosA*-like genes and through the detection of specific point mutations in the associated transporters and regulators involved in uptake.

## RESULTS

All 293 *Enterobacterales* strains were carbapenemase producers: 132/293 produced NDM-type enzymes (111 [84%] E. coli and 21 [16%] *Citrobacter* isolates), 106/293 produced OXA-48-type (76 [72%] E. coli and 30 [28%] *Citrobacter* isolates), 50/293 produced KPC-type enzymes (33 [66%] E. coli and 17 [34%] *Citrobacter* isolates), and 5/293 produced VIM-type enzymes (3 [60%] E. coli and 2 [40%] *Citrobacter* isolates). The agar dilution method (ADM) showed that 71% (208/293) of the isolates retained susceptibility to FOS (MIC ≤ 8 μg/mL) and the remaining 29% (85/293) showed low-susceptibility/resistance profiles. In detail, 41 of 85 (38 [93%] E. coli and 3 [7%] C. freundii isolates) had FOS MICs of 16 μg/mL, 18 of 85 (16 [89%] E. coli and 2 [11%] C. freundii isolates) had FOS MICs of 32 μg/mL, and 26 of 85 (16 [62%] E. coli and 10 [38%] C. freundii isolates) had FOS MICs of ≥64 μg/mL. The sodium phosphonoformate (PPF) test was performed on strains with FOS MICs of ≥64 μg/mL, and only two of 26 FOS-resistant strains were FosA/FosC2 enzyme producers.

PCR investigations of the two strains detected *fosA10* in an NDM-producing strain of E. coli (ECO49406) and a *fosA7* gene in a VIM-producing C. freundii strain (CFR50714). *fosA10* was successfully transferred by conjugation, while attempts to transfer *fosA7* through conjugation and transformation failed.

### *fosA10* in NDM-producing E. coli.

Whole-genome sequencing (WGS) revealed that ECO49406 belonged to sequence type 648 (ST648)/unknown sequence type (Oxford scheme/Pasteur scheme), serotype O102:H6, and the CH type FumC4/FimH27 (fimbrial adhesion gene *fimH* with allele 27 and fumarate hydratase class II gene *fumC* with allele 4). The resistome of ECO49406 consisted of β-lactam (*bla*_NDM-5_), fosfomycin (*fosA10*), aminoglycoside (*aadA2* and *aadA5*), folate pathway antagonist (*dfrA12*, *dfrA17*, and *sul-1*), tetracycline [*tet*(B)], macrolide [*mph*(A)], and quaternary ammonium compound (*qacE*) resistance genes. Moreover, ECO49406 carried a multireplicon IncFIB/FII and an IncK plasmid. Additionally, the virulome consisted of *air*, *chuA*, *eilA*, *fyuA*, *gad*, *irp2*, *iss*, *kpsE*, *kpsMII_K5*, *lpfA*, *ompT*, *terC*, *traT*, and *yfcV* (Table 1).

**TABLE 1:** WGS data for FOS-susceptible and -resistant strains studied^*a*^

Strain	Origin	Location	Species	FOS MIC (μg/mL) and category	FosA variant	Serotype	FumC/FimH	Plasmid	ST (Oxford/Pasteur)	Virulence genes	Other resistance genes	Accession no.
ECO49406	Rectal swab	Prague	E. coli	>128, R	10	O_:H_	4/27	IncK, IncFIB (pB171), IncFII	ST648/unknown	*air*, *chuA*, *eilA*, *fyuA*, *gad*, *irp2*, *iss*, *kpsE*, *kpsMII_K5*, *lpfA*, *ompT*, *terC*, *traT*, *yfcV*	*aadA2*, *aadA5*, *sul-1*, *dfrA12*, *dfrA17*, *tet*(B), *mph*(A), *bla*_NDM-5_, *qacE*	JAPEQT000000000
ECO52246	Urine	Nymburk	E. coli	>128, R	ND	O_:H_	40/30	IncFII(K), IncR	ST131/43	*chuA*, *fyuA*, *gad*, *iha*, *irp2*, *iss*, *kpsE*, *ompT*, *sat*, *sitA*, *terC*, *traT*, *usp*, *yfcV*	*aph(6)-Id*, *aac(6′)-Ib3*, *aph(3′)-Ib*, *aac(6′)-Ib-cr*, *tet*(A), *mph*(A), *qnrB1*, *sul-1*, *sul-2*, *dfrA14*, *ARR-3*, *bla*_OXA-1_, *bla*_TEM-1B_, *bla*_KPC-3_, *qacE*, *catB3*	JAPJVB000000000
ECO52259	Rectal swab	Jičín	E. coli	>128, R	ND	O_:H_	40/30	IncFII(K), IncR	ST131/43	*chuA*, *fyuA*, *gad*, *iha*, *irp2*, *iss*, *kpsE*, *ompT*, *sat*, *sitA*, *terC*, *traT*, *usp*, *yfcV*	*aph(6)-Id*, *aac(6′)-Ib3*, *aph(3′)-Ib*, *aac(6′)-Ib-cr*, *tet*(A), *mph*(A), *qnrB1*, *sul-1*, *sul-2*, *dfrA14*, *ARR-3*, *bla*_OXA-1_, *bla*_TEM-1B_, *bla*_KPC-3_, *qacE*, *catB3*	JAPJVC000000000
ECO52250^*b*^	Urine	Jičín	E. coli	4, S	ND	O_:H_	43/197	IncR	2558/unknown	*chuA*, *clbB*, *cnf1*, *focC*, *fyuA*, *gad*, *hra*, *ibeA*, *iroN*, *irp2*, *iss*, *kpsE*, *kpsMII*, *mchB*, *mchC*, *mchF*, *mcmA*, *ompT*, *papA_F13*, *papC*, *pic*, *sfaD*, *sitA*, *terC*, *usp*, *vat*, *yfcV*	*aac(6′)-Ib3*, *aac(6′)-Ib-cr*, *mph*(A), *sul-1*, *ARR-3*, *bla*_KPC-2_, *bla*_OXA-1_, *qacE*, *catB3*	JAFEXB000000000
ECO52846	Decubitus	Jičín	E. coli	128, R	ND	O_:H_	43/197	IncR	2558/unknown	*chuA*, *clbB*, *cnf1*, *focC*, *fyuA*, *gad*, *hra*, *ibeA*, *iroN*, *irp2*, *iss*, *kpsE*, *kpsMII*, *mchB*, *mchC*, *mchF*, *mcmA*, *ompT*, *papA_F13*, *papC*, *pic*, *sfaD*, *sitA*, *terC*, *usp*, *vat*, *yfcV*	*aac(6′)-Ib3*, *aac(6′)-Ib-cr*, *mph*(A), *sul-1*, *ARR-3*, *bla*_KPC-2_, *bla*_OXA-1_, *qacE*, *catB3*	JAPJVD000000000
ECO53083^*b*^	Stool	Hradec Kralove	E. coli	8, S	ND	O_:H_	40/30	IncFII(k), IncR	ST131/43	*chuA*, *fyuA*, *gad*, *iha*, *irp2*, *iss*, *kpsE*, *ompT*, *sat*, *sitA*, *terC*, *traT*, *usp*, *yfcV*	*aph(3′′)-Ib*, *aac(6′)-Ib3*, *aph(6)-Id*, *aac(6′)-Ib-cr*, *mph*(A), *sul-1*, *sul-2*, *dfrA14*, *ARR-3*, *bla*_KPC-3_, *bla*_OXA-1_, *qacE*, *catB3*	CP070587–CP070593
CFR67526	NA	NA	C. freundii	128, R	ND	NA	NA	IncR, pKPC-CAV1321	ST19	NA	*aac(6′)-Ib-cr*, *mcr-9*, *mph*(A), *sul-1*, *ARR-3*, *bla*_KPC-2_, *bla*_OXA-1_, *bla*_CMY-152_, *qacE*, *catB3*	JAPJVE000000000
CFRC0593595	Urine	Pilsen	C. freundii	128, R	ND	NA	NA	IncFIB, IncFII, IncL	ST98	NA	*aac(6′)-Ib-cr*, *aph(6)-Id*, *aph(3′′)-Ib*, *aac(3′′)-IIa*, *qnrB1*, *sul-1*, *dfrA14*, *tet*(A), *tet*(D), *bla*_OXA-1_, *bla*_OXA-48_, *bla*_CMY-109_, *bla*_CTX-M-15_, *bla*_TEM-1b_, *catB3*	JAPJVF000000000
CFR47654	Urine	Prague	C. freundii	>128, R	ND	NA	NA	IncFIB, IncL	ST98	NA	*aac(6′)-Ib-cr*, *aph(6)-Id*, *aph(3′′)-Ib*, *aac(3′′)-IIa*, *qnrB1*, *sul-1*, *dfrA14*, *tet*(A), *tet*(D), *bla*_OXA-1_, *bla*_OXA-48_, *bla*_CMY-109_, *bla*_CTX-M-15_, *bla*_TEM-1b_, *catB3*	JAPJVG000000000
CFR50714^*c*^	Urine	Prague	C. freundii	64, R	ND	NA	NA	IncFII/IncN	ST673	NA	*aac(6′)-Ib3*, *aph(3′′)-Ib*, *aac(6′)-Ib-cr*, *aadA1*, *fosA7.9*, *qnrS1*, *dfrA14*, *sul-1*, *sul-2*, *bla*_CMY-78_, *bla*_VIM-1_, *qacE*	JAJFDB000000000
CFR47299^*b*^	Rectal swab	Prague	C. freundii	128, R	ND	NA	NA	IncC, IncFII, IncHI2, IncHI2A, IncN	ST65	NA	*aph(6)-Id*, *aph(3′′)-Ib*, *aac(3)-IId*, *aac(6′)-Ib-cr*, *aac(3)-IIa*, *ARR-3*, *qnrA1*, *sul-1*, *sul-2*, *dfrA19*, *tet*(B), *bla*_KPC-2_, *bla*_CMY-48_, *bla*_TEM-1b_, *bla*_OXA-1_, *qacE*, *catB3*, *catA1*	JAFEWM000000000
CFR47462^*b*^	Rectal swab	Prague	C. freundii	128, R	ND	NA	NA	IncFII, IncHI2, IncHI2A, IncN, IncR	ST65	NA	*aph(6)-Id*, *aph(3′′)-Ib*, *aac(3)-IId*, *aac(6′)-Ib-cr*, *aac(3)-IIa*, *ARR-3*, *qnrA1*, *sul-1*, *sul-2*, *dfrA19*, *tet*(B), *bla*_KPC-2_, *bla*_CMY-48_, *bla*_TEM-1b_, *bla*_OXA-1_, *qacE*, *catB3*, *catA1*	JAFEWN000000000
CFR56415^*c*^	Rectal swab	Prague	C. freundii	8, S	ND	NA	NA	IncHI2, IncHI2A	ST95	NA	*aadA1*, *ant(2′′)-Ia*, *aph(3′)-Ia*, *aac(6′)-Ib-cr*, *aadA2b*, *aac(6′)-Ib3*, *mcr-9*, *sul-1*, *dfrA19*, *bla*_VIM-4_, *bla*_CMY-51_, *qacE*, *cmlA1*, *catA2*	CP085726 –CP085728
CFR51929^*c*^	Rectal swab	Prague	C. freundii	16, S	ND	NA	NA	IncHI2, IncHI2A, IncM1	ST95	NA	*aac(6′)-Ib-cr*, *aadA1*, *ant(2′′)-Ia*, *aph(3′)-Ia*, *aac(6′)-Ib3*, *aadA1*, *aadA2b*, *aac(3)-I*, *mcr-9*, *qnrA1*, *sul-1*, *dfrA19*, *tet*(A), *bla*_CMY-51_, *bla*_VIM-4_, *qacE*, *catA2*, *cmlA1*	CP059427–CP059429
CFR47298	Decubitus	Prague	C. freundii	>128, R	ND	NA	NA	IncFIB, IncL	ST98	NA	*aac(3′)-IIa*, *aac(6′)-Ib-cr*, *aph(*6*)-Id*, *aph(3′′)-Ib*, *qnrB1*, *dfrA14*, *sul-2*, *tet*(A), *tet*(D), *bla*_OXA-48_, *bla*_CTX-M-15_, *bla*_TEM-1B_, *bla*_OXA-1_, *bla*_CMY-109_, *catB3*	JARWKP000000000

a NA, not available; ND, not detected; *air*, enteroaggregative immunoglobulin repeat protein; *chuA*, outer membrane hemin receptor; *eilA*, Salmonella HilA homolog; *fyu*, siderophore yersiniabactin receptor; *gad*, glutamate decarboxylase; *irp2*, high-molecular-weight protein 2 nonribosomal peptide synthetase; *iss*, increased serum survival; *kpsE*, capsule polysaccharide export inner membrane protein; *kpsMII_K5*, polysialic acid transport protein; *lpfA*, long polar fimbriae; *ompT*, outer membrane protease; *terC*, tellurium ion resistance protein; *traT*, outer membrane complement resistance; *yfcV*, fimbria-like protein.

b Published in reference 36.

c Included in reference 77.

**FIG 2:**
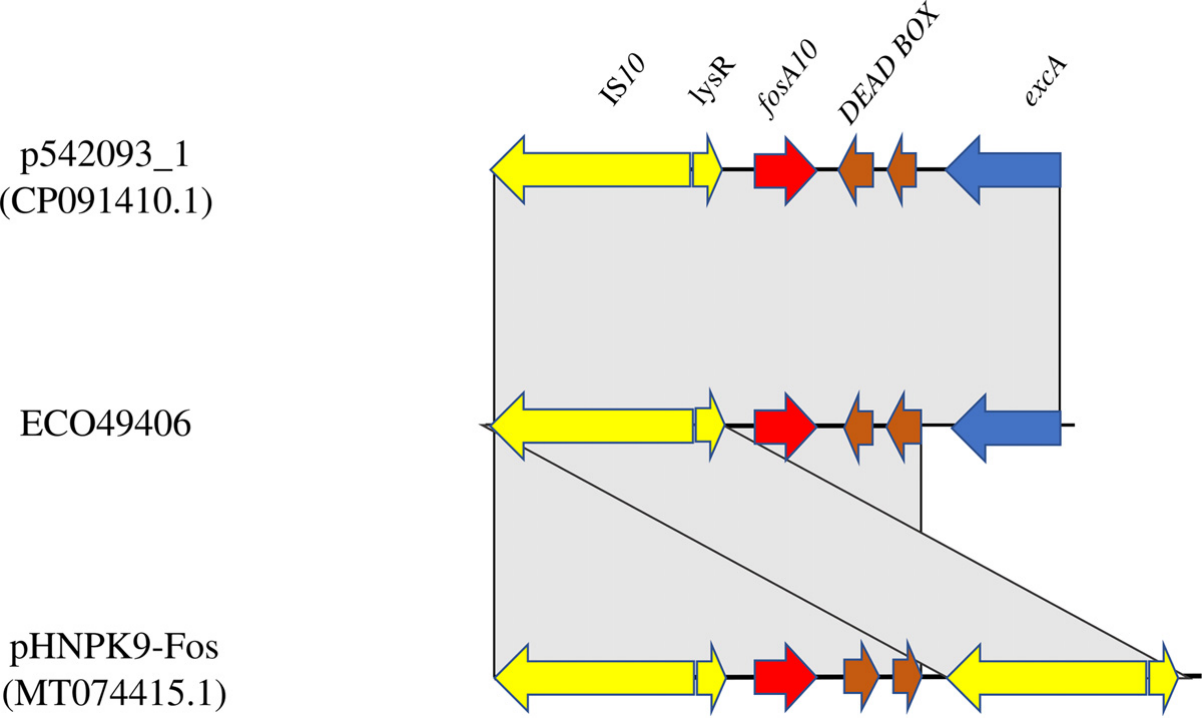
Linear map of the *fosA10* genetic environment of p542093_1, the genetic environment of the *fosA10*-carrying plasmid isolated from ECO49406, and the genetic environment of *fosA10* harbored on an IncFII pHNPK9-Fos. Arrows show the direction of transcription of open reading frames. *excA*, mobile elements, *fosA10*, and other remaining genes are designated by blue, yellow, red, and brown, respectively. Gray shading shows homologous segments with 100% sequence identity.

Based on short-read data and conjugation experiments, *fosA10* was located on the IncK plasmid. *fosA10* was in a genomic cassette of 3,835 bp consisting of *excA-*DEAD box*-fosA10-lysR-*IS*10R.* The BLAST results showed that the cassette shared 100% query and identity with the *fosA10* cassette of the IncB/O/K/Z plasmid p542093_1 (accession no. CP091410.1) and 65% query and 100% identity with the *fosA10* cassette reported in an IncFII plasmid (pHNPK9-Fos; accession no. MT074415.1) (16) collected from veterinary E. coli isolates in China. pHNPK9-*fosA10* cassette (4,328 bp) differed only by (i) containing two copies of IS*10* flanking *fosA10* and the DEAD box and (ii) lacking *excA* (Fig. 2).

### *fosA7.9* in VIM-producing C. freundii.

**FIG 3:**
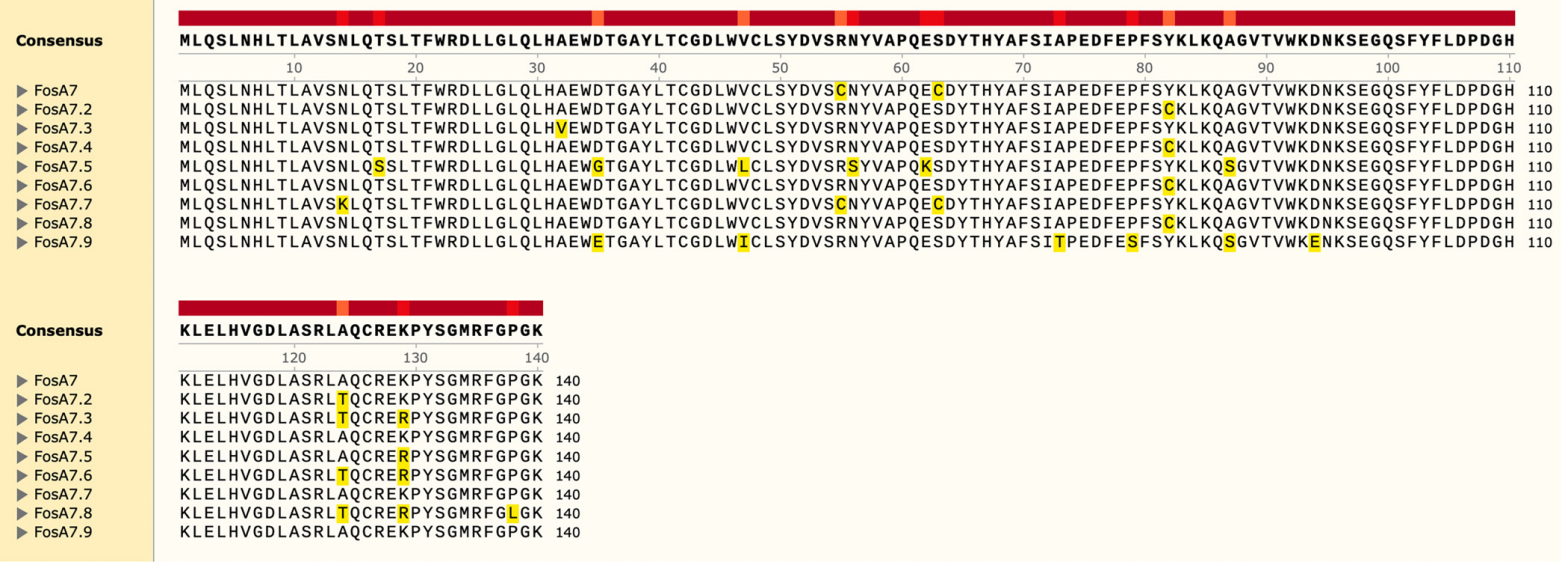
Alignment of amino acid sequences of the FosA7 variants: FosA7 (WP_000941934.1) (94.29% identity with FosA7.9), FosA7.2 (WP_000941935.1) (94.29% identity), FosA7.3 (WP_023231494.1) (93.57% identity), FosA7.4 (WP_023216493.1) (95%), FosA7.5 (WP_000941933.1) (93.57%), FosA7.6 (WP_061377147.1) (93.57%), FosA7.7 (WP_058653118.1) (93.57%), FosA7.8 (WP_079820715.1) (92.86%), and FosA7.9 (UYP40110.1).

**FIG 4:**
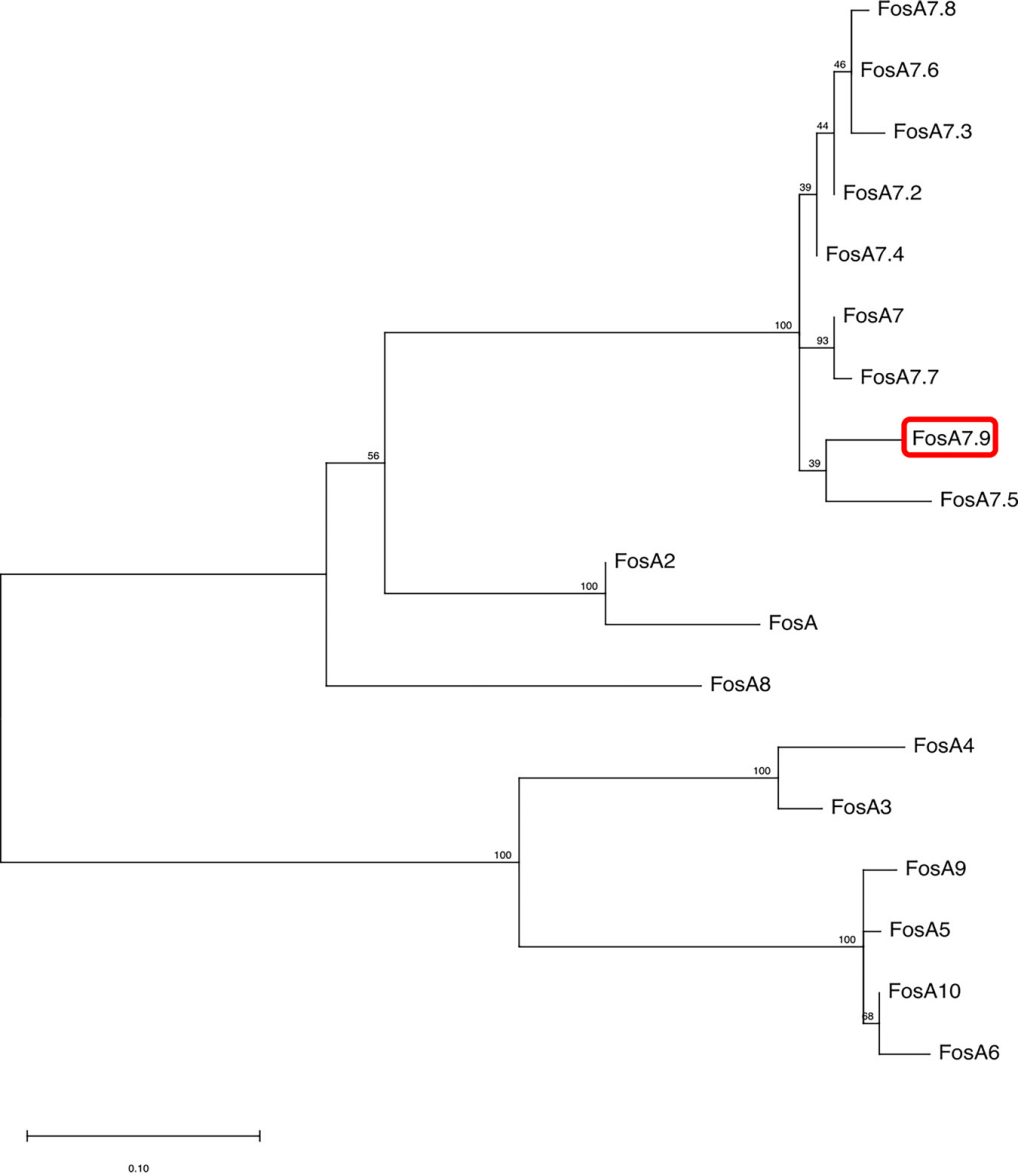
The evolutionary analysis and phylogenetic tree of FosA proteins found in *Enterobacterales* were inferred by using the maximum-likelihood method and JTT matrix-based model using MEGA 11. The tree with the highest log likelihood (−1,275.39) is shown. The percentage of trees in which the associated taxa clustered together is shown above the branches. The tree is drawn to scale. The red rectangle indicates the new FosA7.9 in C. freundii.

**FIG 5:**
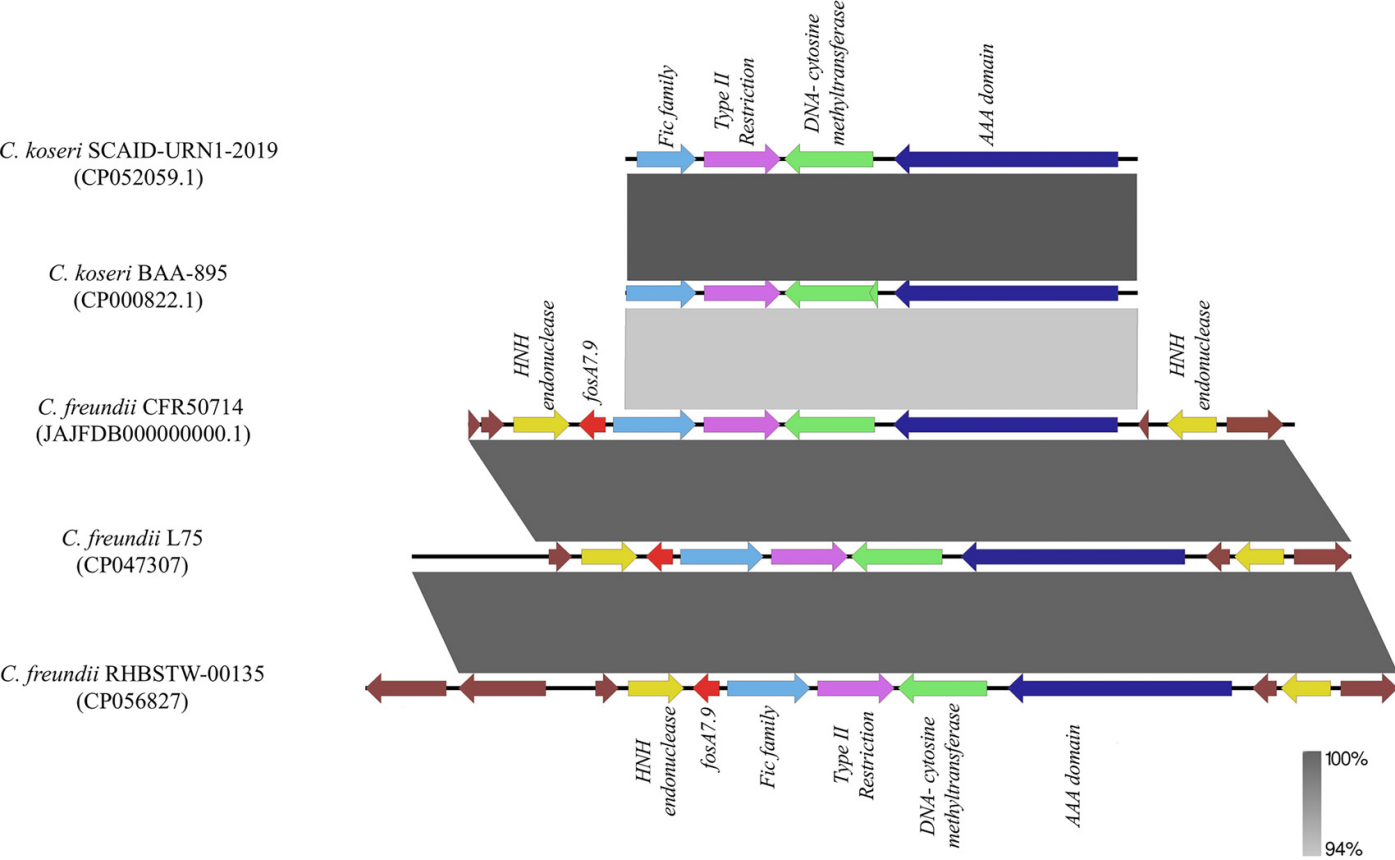
Linear map of the *fic* cassette genetic environment of SCAID-URN1-2019 and BAA-895, the genetic environment of the *fosA7.9* cassette harbored on the CFR50714 chromosome, L75, and RHBSTW-00135. Arrows show the direction of transcription of ORFs. The *fic* family, HNH endonuclease, *fosA7.9*, type II restriction, DNA-cytosine methyltransferase, AAA domain, and other genes are designated by light blue, yellow, red, fuchsia, green, blue and brown, respectively. Homologous segments are indicated with gray shading (light gray, 94% sequence identity; dark gray, 100% identity).

**FIG 6:**
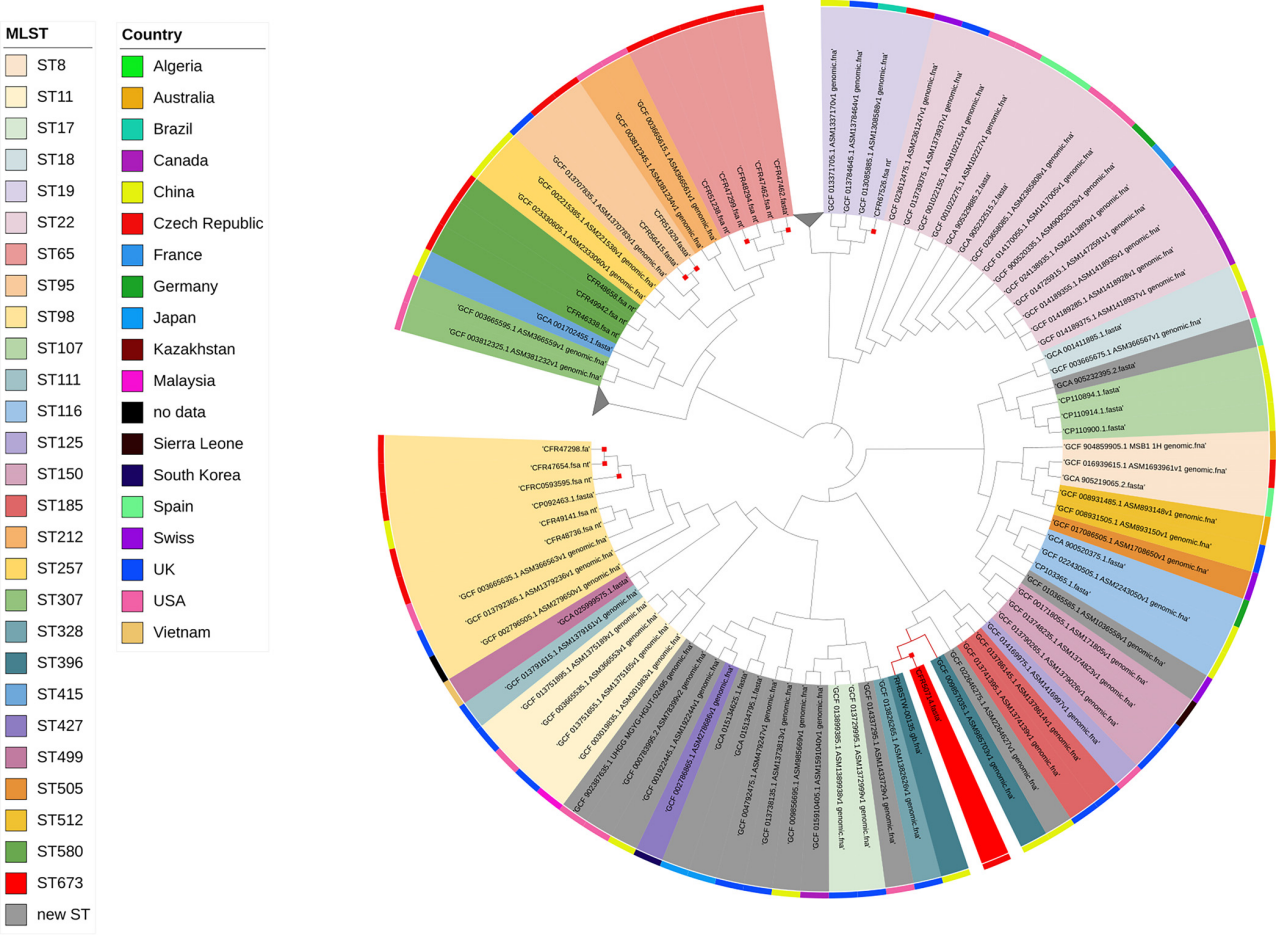
Phylogenetic tree of the nine C. freundii isolates with 103 genomes downloaded from the NCBI database. Red nodes indicate the isolates harboring *fosA7.9* variants. The red square shows the studied isolates. Gray triangles indicate collapsed nodes.

Based on WGS data, C. freundii CFR50714 belonged to ST673 and harbored antibiotic resistance genes for β-lactam (*bla*_VIM-1_ and *bla*_CMY-78_), fosfomycin (*fosA7*), aminoglycoside [*aadA1*, *aac(6′)-Ib3*, and *aac(6′)-Ib-cr*], folate pathway antagonist (*dfrA14*, *sul-1*, and *sul-2*), quinolone (*qnrS1*), macrolide [*mph*(A)] and quaternary ammonium compound (*qacE*). The *fosA7* gene was 423 bp in length and encoded a 140-amino-acid protein. The *fosA7* gene shared highest identity (93.38% identity and 100% query) with the *fosA7.4* variant (accession no. NG_067230.1) and encoded a protein showing 95% identity with FosA7.4 (accession no. WP_023216493.1) and 93.4% with FosA7.5 (Fig. 3 and 4). *fosA7.9* was inserted into a 12,065-bp cassette consisting of the following genes: the HNH endonuclease gene, *fosA7.9*, *fic*, the type II endonuclease restriction gene, the methyltransferase gene, and the HNH endonuclease gene. The BLAST results revealed that the cassette showed similarity with several strains collected worldwide: 100% query and 99.98% identity with the NDM+FosA7.9 coproducer C. freundii L75, collected in China in 2018 from a urine sample (accession no. CP047307) (38), and with the CMY-2+FosA7.9 coproducer C. freundii RHBSTW-00135, collected in 2017 from wastewater influent in the United Kingdom (accession no. CP056827) (39). Additionally, the *fosA7.9* cassette shared 81% query and 94.34% identity with a chromosome cassette of Citrobacter koseri SCAID-URN1-2019 (accession no. CP052059.1) collected in Kazakhstan from a urine sample (40), and with *C. koseri* BAA-895, collected in Maryland from an infant with meningitis (accession no. CP000822.1). Interestingly, the cassette in *C. koseri* lacked the flanking HNH and *fosA7.9* but had an identical *fic* cassette (*fic*-type II endonuclease restriction-methyltransferase) in the same orientation (Fig. 5). The *in silico* analysis and the failed attempts at conjugation suggested a chromosomal location for *fosA7.9*. Sequence data from strain CFR50714, C. freundii L75 (CP047307), and C. freundii RHBSTW-00135 (CP056827) were used to investigate their genomic relatedness to global isolates, to construct a single nucleotide polymorphism (SNP)-based phylogenetic tree. The three aforementioned sequences were compared against 103 genomes found in the NCBI database (Fig. 5). Strain CFR50714 clustered with C. freundii RHBSTW-00135 (ST396) and C. freundii L75 (ST396), forming a clade (Fig. 6).

### Fosfomycin pathway alterations.

**FIG 7:**
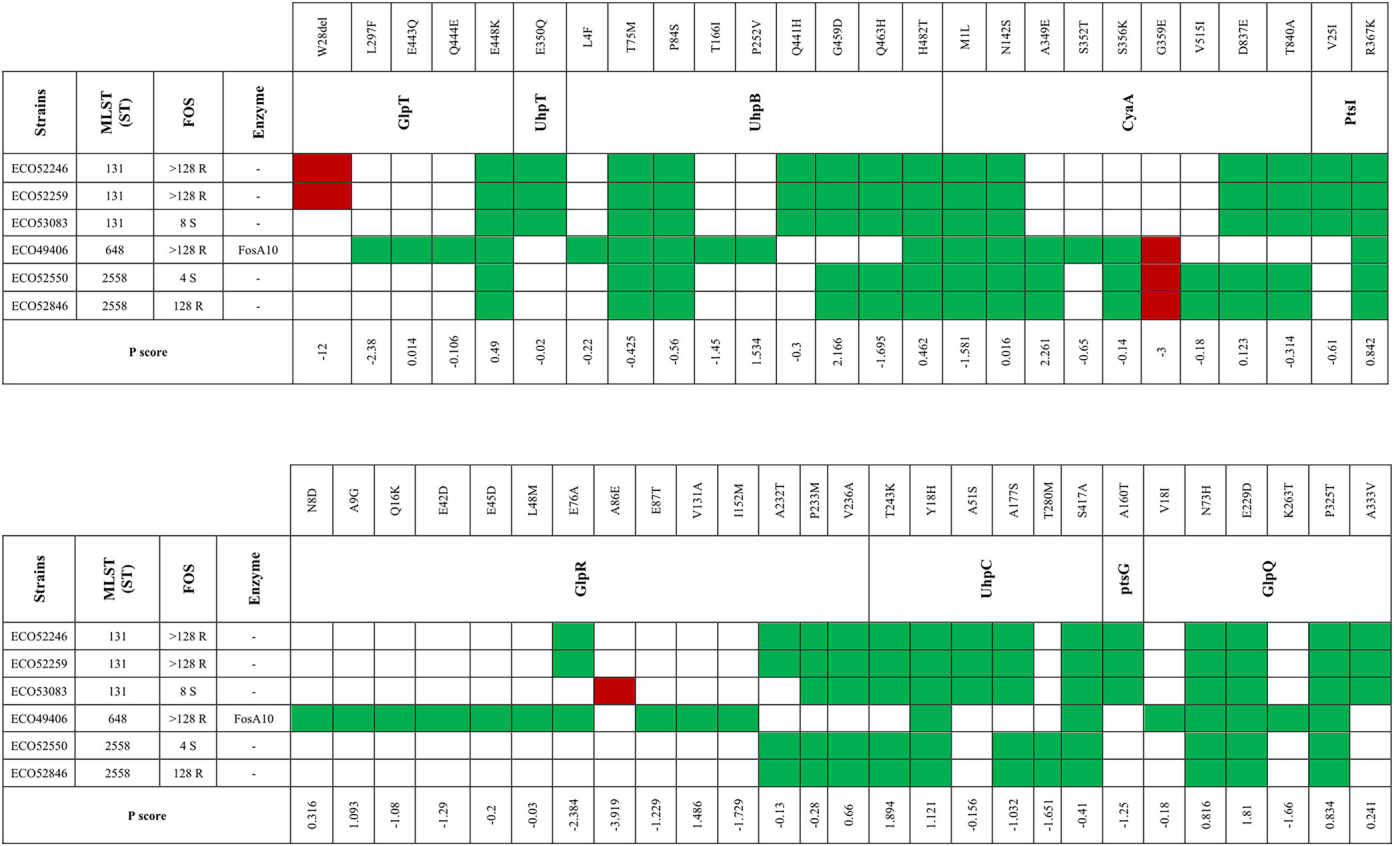
Mutations and P scores obtained by PROVEAN for selected E. coli strains. Red indicates a P score above −2.5 (categorized as deleterious); green indicates a P score below −2.5 (categorized as neutral).

Fifteen sequenced strains were investigated for the presence of point mutations in proteins involved in the FOS pathway. When possible, strains of the same ST but different FOS profiles were compared. Concerning E. coli strains, three of six belonged to ST131, two to ST2558, and one to ST648 (Fig. 7; also, see Fig. S1 in the supplemental material).

All three ST131 E. coli isolates shared neutral substitutions in the amino acids of GlpT, UhpT, UhpB, CyaA, PtsI, GlpR, UhpC, PtsG, and GlpQ (Fig. 7). Additionally, two FOS-resistant (FOS^r^) strains (strains ECO52246 and ECO52259) out of three ST131 strains showed a deleterious W28del (PROVEAN [P] score, −12,042) of GlpT. On the other hand, the remaining FOS-susceptible (FOS^s^) ST131 strain (ECO53083) carried a deleterious substitution (P score, −3.919) in GlpR (A86E) (Fig. 7).

Two E. coli ST2558 strains (FOS^r^ strain ECO528469 and FOS^s^ strain ECO52550) shared the same neutral substitutions in GlpT, UhpB, CyaA, PtsI, GlpR, UhpC, and GlpQ. Moreover, both ST2558 strains showed a deleterious substitution (P score, −3.077) in CyaA (G359E).

The FosA10-producing strain ECO49406 exhibited a wide range of single amino acid substitutions, categorized as neutral, in GlpT, UhpB, CyaA, PtsI, and GlpR. ECO49406 carried the deleterious mutation G359E (P score, −3.077) in CyaA (Fig. 7). No alteration in the target MurA, the transporter UhpT, or the regulators PtsH, UhpA, UhpC, FNR (fumarate and nitrate reduction regulatory protein), and CRR were detected in any of the E. coli strains studied. These results imply a link between ST and mutations in the FOS pathway.

**FIG 8:**
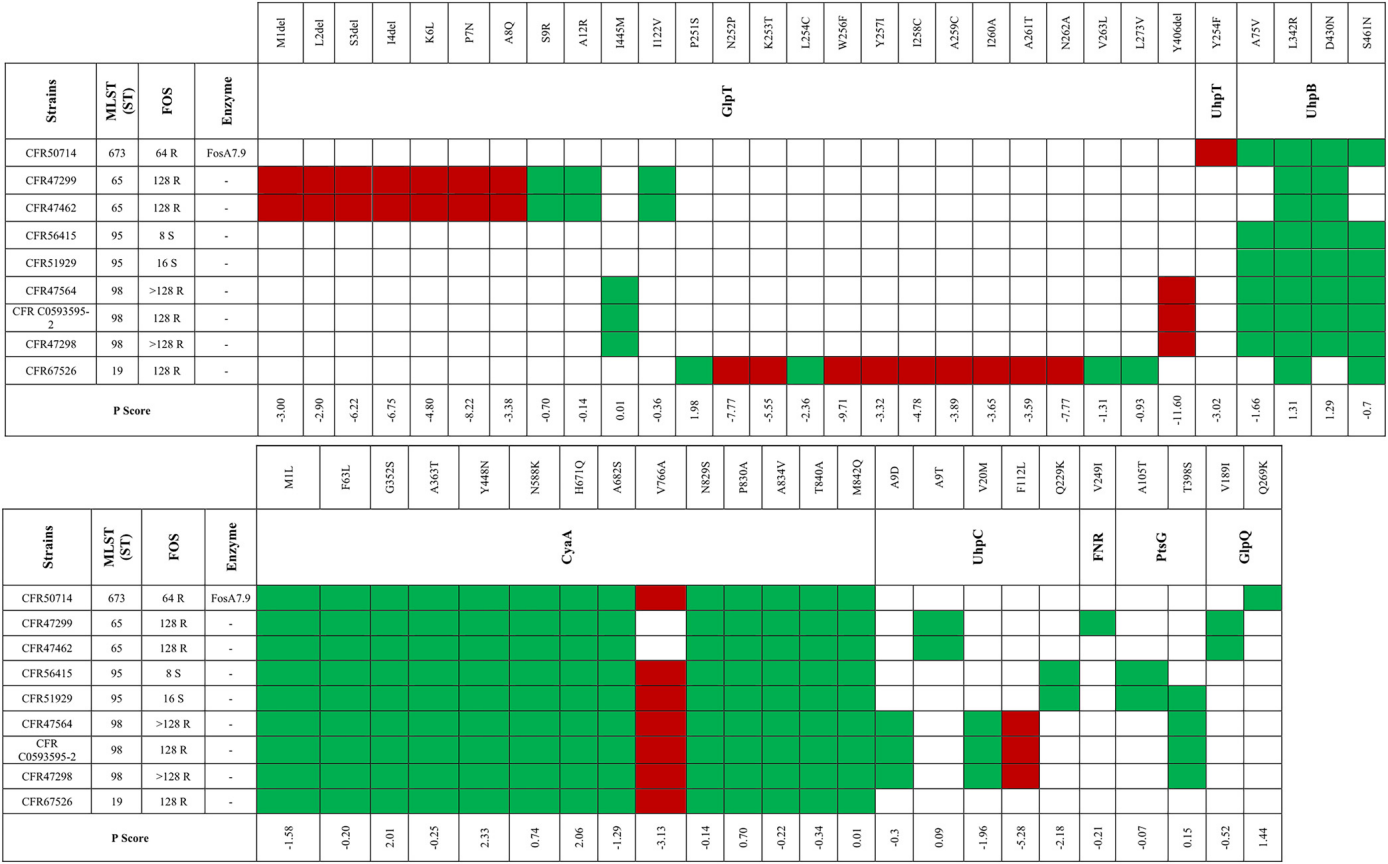
Mutations and P scores obtained by PROVEAN for selected C. freundii strains. Red indicates a P score above −2.5 (categorized as deleterious); green indicates a P score below −2.5 (categorized as neutral).

Regarding C. freundii isolates, three of nine belonged to ST98, two to ST95, two to ST65, one to ST673, and one to ST19. Analysis of the FOS pathway highlighted a similar link between ST and certain amino acid substitutions (Fig. 8). Two FOS^r^ ST65 strains (CFR47299 and CFR47462) shared the same neutral substitutions in GlpT, UhpB, CyaA, UhpC, and GlpQ. Additionally, both isolates missed the first 4 amino acids (aa) (MLSI) and had the deleterious substitutions K6L, P7N, and A8Q (P scores, −4.8, −8.2, and −3.4, respectively) in GlpT. Two FOS^s^ ST95 strains (CFR56415 and CFR51929) accumulated identical neutral substitutions in UhpB, CyaA, UhpC, and PtsG but shared the deleterious mutation V766A in CyaA (P score, −3.13). The same deleterious change in CyaA occurred in strains CFR47298 and CFR50714, belonging to ST98 and ST673, respectively. In contrast, strain CFR47298 had deleterious deletions in GlpT (Y406del; P score, −11.6) and in UhpC (F112L; P score, −5.28). Strain CFR67526, the only ST19 strain, shared the same reported neutral alteration in CyaA. Interestingly, the *glpT* sequence had an insertion of 25 nucleotides (nt) at position 788, leading to a 1,384-nt instead of a 1,359-nt gene. The frameshift mutation affects the amino acids from position 251 to 263aa, which could possibly affect the activity of the transporter (Fig. 8). No alterations in MurA and in the regulators UhpA, PtsH, PtsI, GlpR, CRP, and CRR were detected in any of the C. freundii strains studied. These results together show that the occurrence of mutations at different levels can decrease FOS susceptibility.

## DISCUSSION

Fosfomycin has regained importance in clinical practice and has offered an alternative first-line option against MDR bacterial infections. The pathway of fosfomycin inside bacterial cells depends mainly on GlpT and UhpT activity (2). Acquiring mutations in GlpT and UhpT can impair their transport activity, decreasing FOS uptake into the bacterial cell and hence FOS effectiveness (41–43). However, modifications in these proteins have a high fitness cost, leading to the predominance of FOS-susceptible strains (44).

In the current study, all sequenced E. coli strains had amino acid substitutions in GlpT. The substitutions E448K, Q444E, and E443Q, categorized as neutral by PROVEAN, were reported previously by Takahata et al. and Sorlozano-Puerto et al. and are recognized as not impacting GlpT functionality (3, 9). L297F has been categorized as neutral (P score, −2.375) and was reported previously by Sorlozano-Puerto and colleagues (9); however, the impact on GlpT function has not been investigated yet. Two FOS^r^ E. coli ST131 strains (ECO52246 and ECO52259) contained W28del, classified as deleterious (P score, −12.042). We speculate that there is a possible impact of W28del on GlpT activity, leading to a FOS^r^ profile in E. coli ST131 strains. E. coli ST131 is a hypervirulent and pandemic clone (45) associated with the global spread of ESBLs such as CTX-M-15, KPC-like, and NDM-like enzymes (46–48). The acquisition of additional antimicrobial resistance traits in such successful clones can impact the clinical outcome of infections by such isolates and reduce antibiotic availability.

UhpB is a component of the UhpABC system and is a membrane-associated protein kinase that autophosphorylates and subsequently transfers its phosphate group to UhpA, activating it. CyaA is an adenylate cyclase and catalyzes the formation of cAMP from ATP. UhpB and CyaA exhibited a consistent number of alterations, most of them categorized as neutral. Interestingly, all sequenced E. coli isolates carried the single amino acid substitution M1L (P score, −1.581) but without a clear impact on CyaA functionality. Moreover, three E. coli strains, two FOS^r^ (ECO49406 and ECO52846) and one FOS^s^ (ECO52550), carried the deleterious mutation G359E (P score, −3.077). We hypothesize that this substitution could affect CyaA activity but without leading to a FOS^r^ event at a high level. We hypothesize that in the presence of G359E CyaA, other mechanisms can be involved, leading to the FOS^r^ profiles in these E. coli strains.

GlpR is a repressor of GlpT that binds to G3P, which is essential for *glpT* transcription. In the literature, studies concerning the eventual effect of GlpR on FOS MIC have not been reported yet. Here, we report the deleterious mutation A86E in GlpR. This alteration occurred in a FOS^s^ E. coli ST131 strain (MIC = 8 [susceptible {S}]), and we speculated that there is a possible effect on GlpR activity, leading to the increased FOS MIC. Our findings highlight the possible role that GlpR plays in FOS susceptibility in E. coli strains. Further investigations are needed to understand the nature of the role of GlpR in altering FOS MIC.

In the literature, there are no reports that evaluate modifications in FOS pathway among *Citrobacter* spp.

Here, we describe the first genetic analysis of mutations detected in FOS^s^ and FOS^r^ C. freundii isolates and their effect on FOS susceptibility. In our study, several amino acid substitutions and deletions were detected in GlpT, UhpT, UhpB, CyaA, UhpC, PtsG, and GlpQ. Interestingly, two FOS^r^ C. freundii ST65 isolates lost the first 4 aa of GlpT and carried the deleterious substitutions K6L (P score, −4.8), P7N (P score, −8.22), and A8Q (P score, −3.384). Additionally, both strains lacked the first 18 aa of CyaA. These alterations together could impair the activity of GlpT and CyaA, leading to a high level of FOS^r^. Among the nine C. freundii strains, we detected 30 different single amino acid substitutions in CyaA. Interestingly, all C. freundii isolates except C. freundii ST65 strains had the deleterious mutation V766A in CyaA (P score, −3.126). Regarding the UhpB regulator, all C. freundii harbored the neutral substitutions L342R and D430N and the mutations A75V (P score, −1.66) and S461N (P score, −0.7) (except for C. freundii ST65 strains). Regarding the FOS^r^ C. freundii ST98 strain (CFR 47298), we report the deletion Y406del (P score, −11.6) in GlpT and the deleterious single substitution F112L (P score, −5.28) in UhpC, which could be implicated in FOS resistance. C. freundii ST65 and ST98 are emerging clones involved in the spread of ESBLs. Samuelsen et al. described two cases of CMY-48+OXA-10-coproducing C. freundii ST65 from clinical samples in Denmark, while Schweizer et al. described an outbreak event in Germany, caused by KPC-2-producing C. freundii ST98 isolates (49, 50). The occurrence of deleterious substitutions in proteins implicated in FOS uptake could decrease the effectiveness of FOS and its use against infections by clinically relevant clones such as carbapenemase-producing C. freundii ST98 isolates.

E. coli ST648 is an international high-risk and pathogenic clone, in both clinical and veterinary settings (51). Recently, it has been recognized as a pandemic clone, able to carry carbapenemases such as KPC-2 (52), NDM-1 (53–55), and OXA-48 (53, 55). In 2016, Yang et al. reported an E. coli ST648 strain coproducing NDM-5, CTX-M-55, MCR-1, and FosA3 from a duck in China (56). Here, we report the first case of E. coli ST648 coproducing NDM-5 and FosA10 isolated from humans in the Czech Republic. The genomic environment of *fosA10* showed perfect identity with the *fosA10*-carrying IncI1 plasmid obtained from clinical E. coli ST227 strains in the United Kingdom and close similarity with the *fosA10*-carrying IncFII plasmid from veterinary E. coli ST38 collected in China (57). The global ST648 epidemiology and our findings focus attention on the ease of acquisition of MDR genes in this clone, which could drastically reduce the number of still-active antibiotics. Moreover, the findings highlight (i) the transition of *fosA10* from veterinary to clinical settings, (ii) the ability of the *fosA10* cassette to fit in both the IncFII and IncB/O/K/Z environments, and (iii) the ability of *fosA10*-carrying IncK plasmid to switch from minor to successful clones, such as ST648.

Moreover, we report the first case of a C. freundii ST673 isolate producing VIM and carrying a new FosA7 variant, named FosA7.9. The FosA7.9 gene was inserted in a well-conserved cassette, surrounded by two copies of the HNH endonuclease gene. The HNH endonuclease is a group of homing endonucleases that can act as selfish genetic elements, like transposons, breaking DNA double strands and allowing the acquisition of functional attributes to the host cell, such as antimicrobial resistance (AMR) genes (58, 59). Moreover, the genomic environment of the *fosA7* cassette, flanked by HNH genes, was shared with two clinical C. freundii ST396 isolates collected in China (38). These results and the lack of any insertion elements surrounding *fosA7.9* suggest a possible role for HNH endonucleases in slowly spreading new AMR traits in low-risk and silent hosts, such as C. freundii (35).

### Conclusions.

These results show the emergence of FOS^r^ among CRE from clinical settings in the Czech Republic. The 3-year study revealed a decrease in FOS susceptibility among carbapenemases-producing E. coli and *Citrobacter* strains. In our investigation, the decrease of FOS^s^ could be largely attributable to impairment in GlpT and CyaA activity, significantly reducing the permeability to FOS. To our knowledge, we report the first isolation of FosA10-producing E. coli ST648 and the emergence of a new FosA7 allele, FosA7.9, in C. freundii in the Czech Republic. The emergence and the spread of both chromosome- and plasmid-mediated FOS^r^ mechanisms in CRE could compromise the usefulness of FOS against severe and complex infections. A better knowledge of the genetic mechanisms underlying FOS^r^ may facilitate the creation of rapid DNA-based testing for FOS^r^.

## MATERIALS AND METHODS

### Identification of bacterial isolates, susceptibility determination, and detection of enzymes.

**FIG 9:**
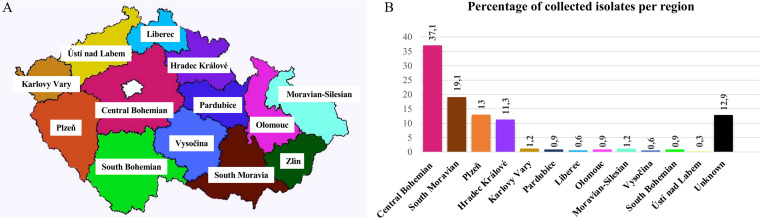
(A) Map of the Czech Republic. (B) Regional percentage of collected isolates (values expressed in percentage).

In the period from December 2018 to February 2022, 223 carbapenemase-producing E. coli isolates and 70 *Citrobacter* isolates (64 C. freundii, 3 Citrobacter amalonaticus, 2 Citrobacter braakii, and 1 Citrobacter youngae isolate) were collected from different health care settings in the Czech Republic and sent to University Hospital in Pilsen, Czech Republic, as part of a national surveillance effort for carbapenemase production in *Enterobacterales* (Fig. 9A and B). Of these, 51.1% (150/293) were collected from urine, 19% (56/293) from rectal swabs, 8% (23/293) from wounds, 8% (23/293) from sputum, 2% (6/293) from blood, and the remaining 11.9% (35/293) from different sources, including stool, pus, bronchoalveolar lavage fluid, decubitus swabs, throat swabs, and aspirates (Fig. S2).

Identification of strains was confirmed by matrix-assisted laser desorption ionization–time of flight mass spectrometry (MALDI-TOF MS) with MALDI Biotyper software (Bruker Daltonics, Bremen, Germany). The production of carbapenemases (metallo-β-lactamase, OXA-48, and KPC) was assessed with the double-disc synergy test with EDTA, the temocillin disc test, and the phenylboronic acid test (37). FOS MICs were evaluated using ADM and interpreted according to EUCAST clinical breakpoints (v 12.0) and guidelines. Production of FosA-like and FosC2 enzymes was detected by the PPF test (60).

### Molecular investigations.

All the isolates were screened by PCR for the presence of *bla*_KPC-like_, *bla*_NDM-like_, *bla*_VIM-like_, and *bla*_OXA-48-like_ genes, as reported elsewhere (36). Positive strains with PPF test were additionally screened by PCR for the presence of *fosA*, *fosA2*, *fosA3*, *fosA4*, *fosA5*, *fosA6*, *fosA7*, *fosA10*, and *fosC2* (15, 16, 21, 61).

### WGS and analysis.

A total of 15 strains (6 E. coli and 9 C. freundii strains) were selected as representative for genomic content and FOS pathway mutation analysis. The selection was based on FOS MICs: the selection included 11 strains (four E. coli and seven C. freundii strains) with FOS MICs of ≥64 μg/mL (resistant [R]) and four strains (two E. coli and two C. freundii strains) with FOS MICs of ≤16 μg/mL (S). The four FOS-susceptible strains were included to compare differences in FOS resistance (FOS^r^) and FOS susceptibility (FOS^s^) profiles. In detail, the selection for FOS^s^ E. coli included FOS MICs corresponding to the FOS epidemiological cutoff (ECOFF) value and one dilution beyond the FOS ECOFF value according to EUCAST (https://mic.eucast.org/search/?search%5Bmethod%5D=mic&search%5Bantibiotic%5D=100&search%5Bspecies%5D=-1&search%5Bdisk_content%5D=-1&search%5Blimit%5D=50). For FOS^s^ C. freundii isolates, FOS ECOFF values are not available; thus, low-level susceptibility profiles for FOS were selected (FOS MIC = 8 μg/mL and 16 μg/mL).

Genomic DNA was extracted using a NucleoSpin microbial DNA kit (Macherey-Nagel, Germany). WGS was performed on seven selected strains with the NovaSeq 6000 system with a 2 × 250 paired-end run following Nextera XT library preparation (Illumina Inc., San Diego, CA, USA). The remaining eight strains belonged to two different projects (Table 1), and WGS was previously performed with both the Illumina MiSeq platform (Illumina Inc., San Diego, CA, USA) and the Sequel I platform (Pacific Biosciences, Menlo Park, CA, USA) (36). Reads were assembled using SPAdes software (62). Assembled sequences were annotated using the RAST (Rapid Annotation using Subsystems Technology) server (63). The resistome, plasmid replicons, mobile elements, multilocus sequence types (MLST), and plasmid MLST (pMLST) were determined by uploading the assembled sequences to ResFinder 4.1 and CARD (64, 65), PlasmidFinder (66), ISfinder (67), MLST 2.0 (68), and pMLST 2.0 (66), respectively. Comparative genome alignment was done using Mauve v.2.4.0 and SnapGene (SnapGene Software). A linear map of chromosomal environments was created by using EasyFig (69) and the graphic editor Procreate (Savage Interactive, Tasmania, Australia).

### Phylogenetic analysis.

Phylogenetic relationships between the selected sequenced isolates and global genomes were investigated. Phylogenetic trees were obtained using core genome, recombination, and SNPs by using parsnp v1.2, available in the harvest suite (70), and using a corresponding reference genome. Graphic illustration of the trees was build with the Interactive Tree Of Life (iTOL) (https://itol.embl.de/) (71). For the construction of the SNP-based phylogenies, 160 Escherichia coli genomes and 112 Citrobacter freundii genomes were downloaded from the NCBI assembly database, including complete and draft genomes. E. coli ECO49406 and C. freundii RHBSTW-00135 were use as respective references. The evolutionary analysis of FosA-like proteins in *Enterobacterales* was conducted by MEGA 11 (72), using the maximum-likelihood method and the Jones-Taylor-Thornton (JTT) matrix-based model (73).

### Conjugation/transformation assay.

The conjugal transfer of *fosA* genes was tested in liquid medium using the E. coli A15 strain (Azd^r^) as a recipient. Transconjugants were selected on MacConkey agar (Scharlab, SL, Barcelona, Spain) plates containing sodium azide (100 mg/L) (Sigma-Aldrich, St. Louis, MO, USA), FOS (64 mg/L) (Sigma-Aldrich), and G6P (25 mg/L) (Roche). The presence of *fosA*-like genes and the plasmid content in transconjugants were further confirmed by PCR and PCR replicon typing (PBRT 2.0 kit), respectively (74). Since *fosA7* was not transferable by conjugation, transformation was carried out with CFR50714; plasmid extraction was performed using a Qiagen maxi kit (Qiagen, Hilden, Germany), and competent E. coli Top10 cells were used as the recipient. Transformants were selected on Mueller-Hinton (MH) agar (Oxoid, Hampshire, UK) with 32 mg/L FOS (Sigma-Aldrich) and 25 mg/L G6P (Roche) (75).

### Protein mutations.

The effects of amino acid alterations on the biological function were predicted using the online PROVEAN (Protein Variation Effect Analyzer) platform (http://provean.jcvi.org/index.php) (76). PROVEAN predicts protein sequence variations, including single or multiple amino acid substitutions, insertions, or deletions. The platform produces a delta alignment score based on the reference and variant versions of a protein query sequence with respect to sequence homologs collected from the NCBI protein database through BLAST. For each substitution, the tool provides a score (P score) categorized in three classes: (i) if the P score is equal to or below the cutoff of −2.5, the protein alteration is categorized as deleterious (potential loss of protein structure or function); (ii) if the P score is above the threshold, the alteration is marked as neutral (no alteration in the structure or function of the protein) (9). Amino acid variations in MurA, GlpT, UhpT, UhpA, UhpB, UhpC, CyaA, PtsI, PtsH (phosphocarrier protein HPr), GlpR, CRP, CRR (enzyme IIA [Glc]), PtsG (phosphotransferase system [PTS] glucose-specific EIICB component), FNR (fumarate and nitrate reduction regulatory protein), and GlpQ (glycerophosphodiester phosphodiesterase) were investigated (accession numbers are reported in Table S1).

### Data availability.

The nucleotide sequence of *fosA7.9* has been uploaded to GenBank under the accession number ON245013. GenBank accession numbers of the sequenced strains are presented in Table 1.
